# *Mycoplasma genitalium* Protein of Adhesion Promotes the Early Proliferation of Human Urothelial Cells by Interacting with RPL35

**DOI:** 10.3390/pathogens10111449

**Published:** 2021-11-08

**Authors:** Pei Dai, Xiangying Deng, Peng Liu, Lingling Li, Dan Luo, Yating Liao, Yanhua Zeng

**Affiliations:** Hunan Provincial Key Laboratory for Special Pathogens Prevention and Control, Hunan Province Cooperative Innovation Center for Molecular Target New Drug Study, Institute of Pathogenic Biology, Hengyang Medical College, University of South China, Hengyang 421001, China; 20202013110899@stu.usc.edu.cn (P.D.); xiangyingbszn@163.com (X.D.); pengliu@live.cn (P.L.); 20202013110964@stu.usc.edu.cn (L.L.); 20192013110765@stu.usc.edu.cn (D.L.); liaoyating@stu.usc.edu.cn (Y.L.)

**Keywords:** *Mycoplasma genitalium*, MgPa, T7 phage-displayed cDNA library, interacting proteins, RPL35

## Abstract

*Mycoplasma genitalium* is a newly recognized pathogen associated with sexually transmitted diseases (STDs). MgPa, the adhesion protein of *Mycoplasma genitalium*, is the main adhesin and the key factor for *M. genitalium* interacting with host cells. Currently, the long-term survival mechanism of *M. genitalium* in the host is not clear. In this study, a T7 phage-displayed human urothelial cell (SV-HUC-1) cDNA library was constructed, and the interaction of MgPa was screened from this library using the recombinant MgPa (rMgPa) as a target molecule. We verified that 60S ribosomal protein L35 (RPL35) can interact with MgPa using far-Western blot and co-localization analysis. According to the results of tandem mass tag (TMT) labeling and proteome quantitative analysis, there were altogether 407 differentially expressed proteins between the pcDNA3.1(+)/MgPa-transfected cells and non-transfected cells, of which there were 6 downregulated proteins and 401 upregulated proteins. The results of qRT-PCR demonstrated that interaction between rMgPa and RPL35 could promote the expressions of EIF2, SRP68, SERBP1, RPL35A, EGF, and TGF-β. 3-(4,5)-Dimethylthiahiazo(-z-y1)-3,5-di-phenytetrazoliumromide bromide (MTT) assays corroborated that the interaction between rMgPa and RPL35 could promote SV-HUC-1 cell proliferation. Therefore, our findings indicated that the interaction between rMgPa and RPL35 can enhance the expressions of transcription-initiation and translation-related proteins and thus promote cell proliferation. This study elucidates a new biological function of MgPa and can explain this new mechanism of *M. genitalium* in the host.

## 1. Introduction

*Mycoplasma genitalium* is an emerging pathogen causing genitourinary tract infections in both men and women. The bacterium is reportedly linked to male urethritis and adverse reproductive sequelae in women, including cervicitis, endometritis, pelvic inflammatory diseases, and nongonococcal urethritis [1]. Researchers have proven that *M. genitalium* is closely associated with human immunodeficiency virus (HIV) infection and can promote the replication of HIV after invading host cells [2,3,4]. *M. genitalium* can also combine with motile male sperm and, thus, lead to diffuse infection, which causes reproductive diseases and infertility [5].

The initial adhesion of *M. genitalium* to host cells is considered to be essential for the colonization of *M. genitalium,* leading subsequently to human urethritis [6]. The *M. genitalium* protein of adhesion, MgPa, is a key adhesion component located on the surface of the terminal organelle of *M. genitalium* and plays vital roles in the pathogen’s adhesion to and invasion of host cells [7]. The adhesion of *M. genitalium* to host cells also requires the participation of some auxiliary proteins, such as P32, P114, P110, and MG218. When these proteins are absent, the MgPa cannot be located at the terminal organelle, thus affecting the adhesion and infectivity of the *M. genitalium* to host cells [8].

*M. genitalium*’s infection or invasion of host cells is carried out through the process of interaction between *M. genitalium* and host cells. *M. genitalium* adhesion proteins are multifunctional proteins, in addition to their critical role in cell adhesion, they are important for locomotion and cell invasion [9,10,11,12,13,14,15,16,17,18,19,20]. Therefore, adhesion proteins are virulence factors that are essential for colonization, communication, and persistent infection of *M. genitalium*. McGowin et al.’s findings suggested that *M. genitalium* invades host cells rapidly, utilizing intracellular survival niches to evade the effective cellular immune responses of the host [11]. However, the mechanism of primary infection and persistence of *M. genitalium* in host tissues is unclear. 

Ribosome, the major organelles involved in protein biosynthesis, participate mainly in the synthetic process involving the translation of mRNA into protein [12]. Mature ribosomes consist of a large subunit and a small subunit, and each subunit consists of one or more ribosomal (rRNA) molecules and ribosomal proteins (RPs). RP is the main component of ribosomes and plays a significant role in the biosynthesis of intracellular proteins [13]. Previous studies confirmed that RPs not only participate in the biosynthesis of ribosomes but also play independent roles in protein biosynthesis processes, such as cell proliferation, apoptosis and differentiation, and the translation of mitochondrial proteins [14]. The 60S ribosomal protein L35 (RPL35) is an important component of a ribosomal subunit and plays a central role in protein translation and endoplasmic reticulum docking [15]. 

In our previous study, the soluble recombinant MgPa (rMgPa) containing the dominant epitope (1075–1364 aa) with strong immunogenicity and low homology with *M. pneumoniae* adhesion protein P1 was expressed and purified [16]. In the present study, a T7 phage-displayed human urothelial cell (SV-HUC-1) cDNA library was constructed to screen the interacting protein of rMgPa. The interaction between MgPa and the interacting protein was proven in vitro and intracellularly using far-Western blotting and co-location analysis. The effects of the interaction between rMgPa and the interacting protein on the function of SV-HUC-1 cells was analyzed through TMT protein quantitative analysis. Our study is aimed at further understanding the interaction between *M. genitalium* and the host to provide an experimental basis for the possible survival strategy of *M. genitalium.*

## 2. Results

### 2.1. The T7 Phage-Displayed cDNA Library of SV-HUC-1 Cells Was Successfully Constructed

**Figure 1:**
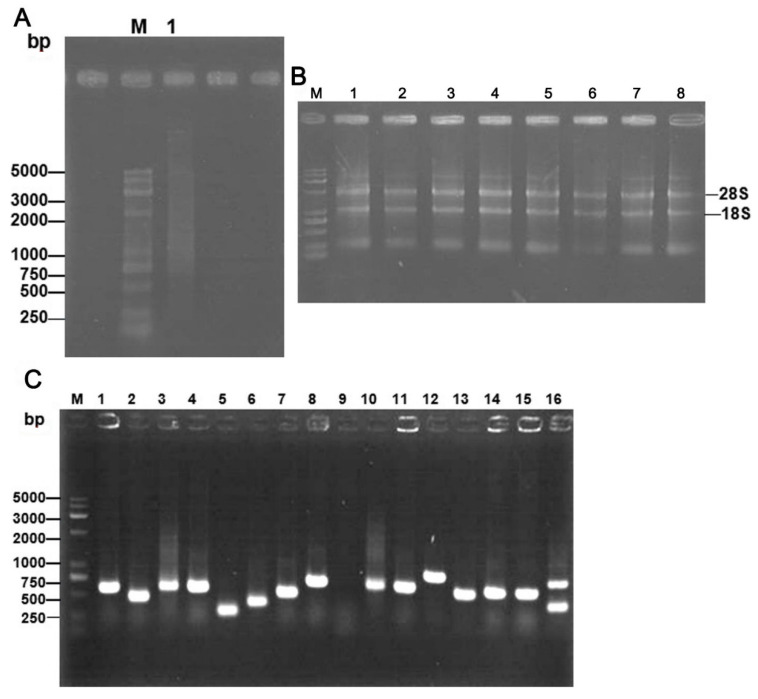
Construction of T7 phage-displayed cDNA library of SV-HUC-1 cells. (**A**) Electropherogram of ds cDNA extracted from SV-HUC-1 cells. (**B**) Agarose gel electropherogram of total RNA from SV-HUC-1 cells. M: DNA marker DL5000. 1: ds cDNA. (**C**) PCR products of random clones of the cDNA library were detected by agarose gel electrophoresis. M: DNA marker 1–26. Numbers of different random clones.

The T7 phage-displayed cDNA library of SV-HUC-1 cells was constructed in order to screen the rMgPa-interacting proteins from the human urethral epithelium. Total RNA was extracted from SV-HUC-1 cells. The results showed that the ds-cDNA was synthesized by reverse transcription and analyzed by agarose electrophoresis (Figure 1A). Then, the bands of 28S and 18S RNA were clear (Figure 1B), which indicated that the extracted total RNA has good quality. The results showed that mRNA with high purity was isolated, and the ds-cDNA fragment could be used for further experiments. Fifteen plaques selected randomly were successfully amplified by PCR (Figure 1C), which demonstrated that the recombination rate of the library reached 93.75% (15/16). The exogenous DNA fragments inserted into phages were all within the range of 250–1000 bp, which demonstrated that the library has good diversity. Results from BLAST analysis revealed that these inserted sequences were all derived from the human genome DNA, which indicated that the cDNA library of SV-HUC-1 cells was constructed successfully. The SV-HUC-1 T7 phage-display cDNA library constructed by our research group was amplified in the host strain BLT5403 (Supplementary Materials Figure S1). The library was used subsequently for biopanning in this study. 

### 2.2. rMgPa-Specific T7 Phages Were Enriched Successfully

First, we conducted four rounds of affinity biopanning, the phage yield ranged from 0.67 × 10^−4^ to 1.7 × 10^−3^, the results showed that the phage with specific binding to rMgPa was significantly enriched (Table 1). In order to analyze the foreign DNA sequences inserted into T7 bacteriophages, the PCR amplification products from positive plaques were sequenced and then analyzed by BLAST alignment. Agarose gel electrophoresis analysis of PCR products of the displayed phages are shown in Supplementary Materials Figure S2. The results showed that there were 7 different sequences (named representative phages P1–P7) for all 32 phages that displayed exogenous sequences. The occurrence frequency of P1 was the highest (62.5%), and the 455 bp base pair inserted P1 was 99% homologous with the 21st–475th DNA sequence of the RPL35 gene. The protein ID, coded protein, matched sequence range, homology, and repeated times of representative phages are listed in Table 2. These results indicated that the seven proteins might be rMgPa-specific binding proteins, and RPL35 might be a dominant rMgPa-interacting protein challenged from the T7 phage-displayed cDNA library of SV-HUC-1 cells. The result of the agarose gel electrophoresis analysis of PCR products for partial phages is shown in Supplementary Materials Figure S2.

### 2.3. The Representative Phages Could Combine Specially with rMgPa 

**Figure 2:**
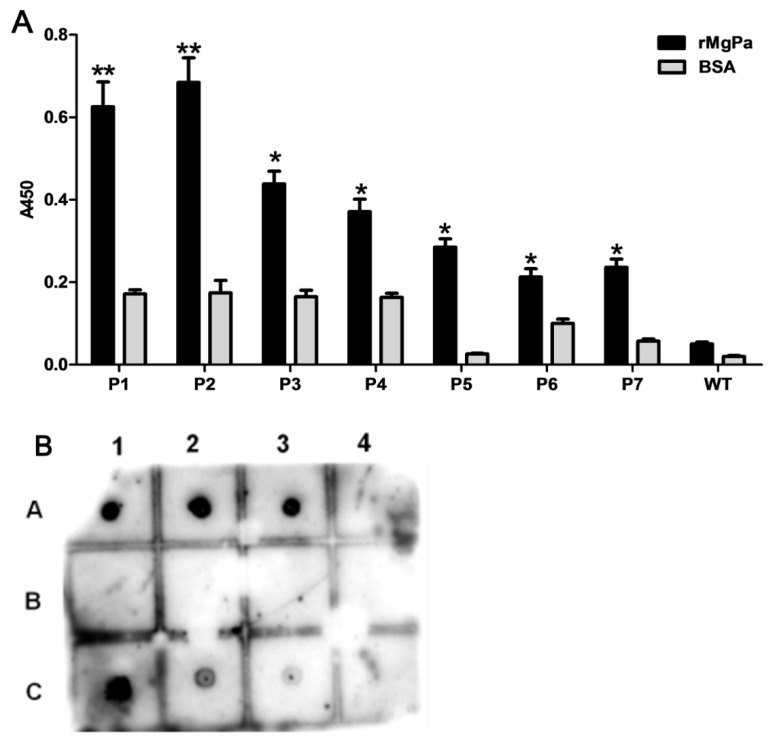
rMgPa-specific T7 phages were enriched successfully. (**A**) The specific combinations between representative phages and rMgPa were detected by ELISA. Note: P1–P7: number of representative phages. WT: wildtype phage. BSA was used as blank control. The note ** indicates *p* < 0.01 compared with the BSA group, * indicates *p* < 0.05 compared with the BSA group. (**B**) The specific combinations between representative phages and rMgPa were detected by dot immunobinding. Note: A1–A4: representative phages P1–P4; B1: WT control; B2: BSA control; B3–B4: blank control; C1: rMgPa positive control; C2–C4: representative phages P5–P7.

Indirect ELISA was performed to verify whether the representative phages could combine specific with rMgPa. Results showed that the optical density (OD) values of the seven representative phages were much higher than that of the bovine serum albumin (BSA) control group (*p* < 0.05), which demonstrated that these representative phages could bind specially to rMgPa (Figure 2A). To further verify whether the representative phages were rMgPa specific, the dot immunobinding assay was used to detect the interaction between rMgPa and these representative phages. As shown in Figure 2B, representative phages and positive control groups showed clear spots, while no spots appeared for the control group. These results confirmed those of the ELISA. In addition, these results indicate that the seven representative phages could specifically combine with rMgPa.

### 2.4. rMgPa Interacts with RPL35

**Figure 3:**
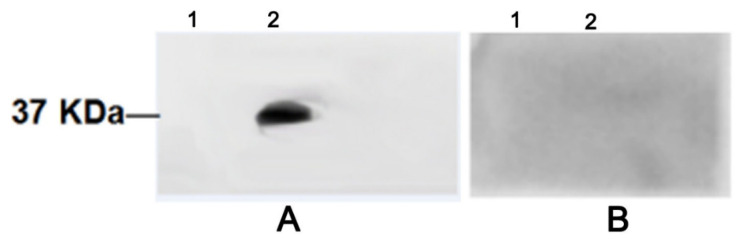
The interaction between rMgPa and RPL35 was detected by far-Western blot. rMgPa was incubated with total cell proteins and then with anti-RPL35 antibody and HRP-conjugated goat anti-rabbit IgG antibody, and there was a distinct band at 37 kDa, whereas no band was observed for the control group (in which rMgPa was incubated directly with anti-RPL35 antibody). Note for (**A**) the membrane was incubated with total cell proteins and for (**B**) it was not. Lane1: no rMgPa (blank control); 2: rMgPa.

The eukaryotic expression vector of rMgPa was constructed, and the expression of rMgPa into cells was detected by Western blot (Supplementary Materials Figures S3 and S4). Far-Western blotting was performed to confirm whether RPL35 could interact with rMgPa. As shown in Figure 3, there were bands of about 37 kDa in every group, except for the negative and blank control, which suggested that RPL35 could interact with rMgPa in vitro.

### 2.5. rMgPa and RPL35 Could Interact in SV-HUC-1 Cells

**Figure 4:**
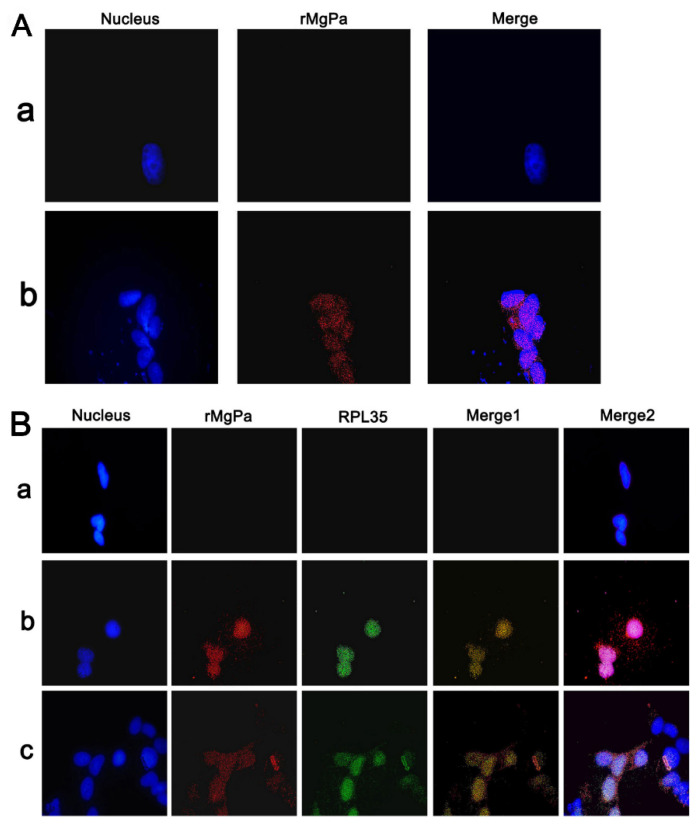
rMgPa and RPL35 could interact in SV-HUC-1 cells. (**A**) The pcDNA3.1(+)/MgPa-transfected SV-HUC-1 cells were detected using indirect immunofluorescence (100×). SV-HUC-1 cells were fixed, blocked, and incubated with anti-MgPa antibody followed by incubation with Cy3-conjugated AffiniPure goat anti-rabbit IgG (H + G), then the cells were incubated with DAPI staining solution (DAPI). Note: a: control; b: pcDNA3.1(+)/MgPa groups. Representative images are shown. (**B**) Co-location of RPL35 and rMgPa in SV-HUC-1 cells (100×). Transfection of different concentrations of pcDNA3.1(+)/MgPa. The cells were prepared and incubated with the primary antibody mixture containing rabbit anti-rMgPa antibody and mouse anti-RPL35 antibody, then incubated with the second antibody mixture containing FITC-conjugated AffiniPure goat anti-mouse IgG(H + G) and TRITC-conjugated AffiniPure goat anti-rabbit IgG(H + L), the cells were then incubated with DAPI. Note: a: control; b: 6 μg pcDNA3.1(+)/MgPa; c: 4 μg pcDNA3.1(+)/MgPa; nucleus (blue), rMgPa (red), RPL35 (green). Merge 1 was composed of red (rMgPa) and green (RPL35), Merge 2 was composed of red (rMgPa), green (RPL35), and blue (nucleus). Representative images are shown.

Co-localization assays were performed to determine whether RPL35 could interact with rMgPa in SV-HUC-1 cells. Firstly, we observed, after indirect immunofluorescence, that the pcDNA3.1(+)/rMgPa vector was transfected successfully into SV-HUC-1 cells (Figure 4A). The results of a double immunofluorescence staining in SV-HUC-1 cells showed that the immunoreactivities between RPL35 and rMgPa were distributed mainly in the cytoplasm and nuclear region (Figure 4B). These results indicate that rMgPa could interact with RPL35 intracellularly.

### 2.6. Differential Proteins Were Identified Successfully by TMT Analysis

**Figure 5:**
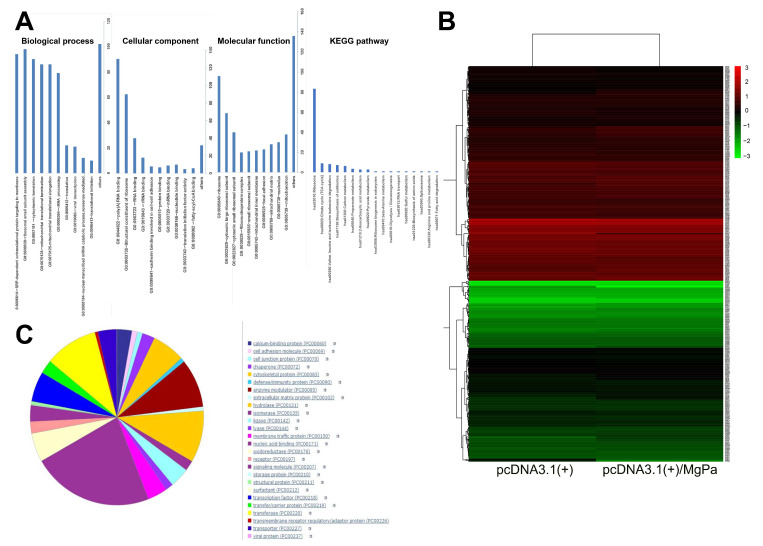
Differential protein analysis. The transfection group and the empty-plasmid group were established. Gene ontology of differential proteins. (**A**) GO enrichment analysis. (**B**) Thermal analysis of differential proteins. (**C**) PANTHER protein class categories of total DEGs.

To further explore the effect of the rMgPa and RPL35 interaction on cell function, the TMT protein quantitative assay was implemented to screen the differential proteins before and after pcDNA3.1(+)/MgPa was transfected into SV-HUC-1 cells. A total of 3843 proteins were identified, of which 407 proteins were expressed differentially. Compared with the empty-plasmid-transfected group, 6 proteins were downregulated, and 401 proteins were upregulated for the pcDNA3.1(+)/MgPa-transfected group. As far as the biological process was concerned, these proteins were involved mainly in the co-translation of the signal recognition particle subunit (SRP), translation initiation, and transcription of mRNA by GO enrichment analysis (Figure 5A). Regarding the cellular composition, these proteins were mainly related to ribosomes. In terms of molecular function, these differential proteins were mainly related to the binding and structural composition of RNA. The enrichment of differential protein in the KEGG pathway showed that it was mainly concentrated in the ribosomal signaling pathway and citrate cycle. The clustering of differentially expressed proteins in the control and the transfection group is shown in Figure 5B, where red represents upregulated proteins, green represents downregulated proteins. The differentially expressed proteins were mainly related to cell adhesion, intercellular junction, and defensive and immune-related proteins, as shown in Figure 5C.

### 2.7. The mRNA Expressions of Representative Factors Were Increased

**Figure 6:**
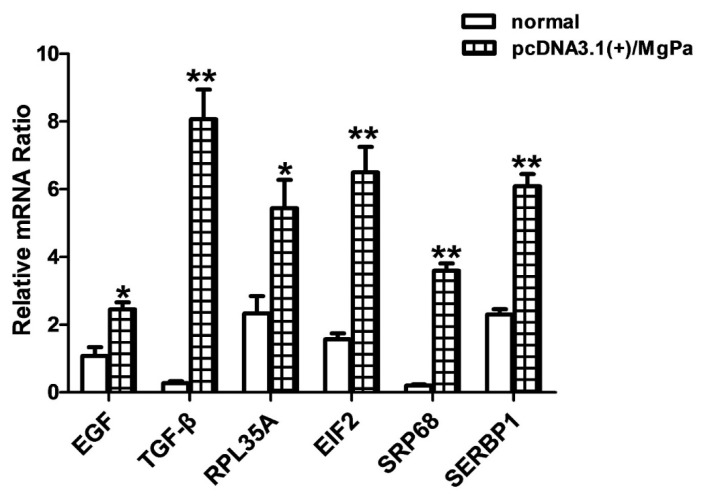
The mRNA expressions of representational factors were verified by RT-qPCR (*n* = 3). The mRNA expressions of representative differential proteins in the empty-plasmid group and the transfection group were detected by RT-qPCR. As shown in the figure, the expression of mRNA the transfection group was significantly higher than in the normal group. Note: * represents *p* < 0.05 and ** represents *p* < 0.01.

Representative factors related to co-translation, translation initiation, ribosomal assembly, and RNA transcription, such as EIF2, SRP68, SERBP1, RPL35A, EGF, and TGF-β were selected for the analysis of their gene expression levels using RT-qPCR. As shown in Figure 6, the expression of all these genes increased to some extent after pcDNA3.1(+)/MgPa was transfected into SV-HUC-1 cells, and TGF-β, RPL35A, and SRP68 expressions were increased significantly. These results were consistent with those from the TMT analysis. All these findings showed that the interaction between rMgPa and RPL35 could regulate the transcription and translation of these proteins. 

### 2.8. The Interaction between rMgPa and RPL35 Can Promote Cell Proliferation

**Figure 7:**
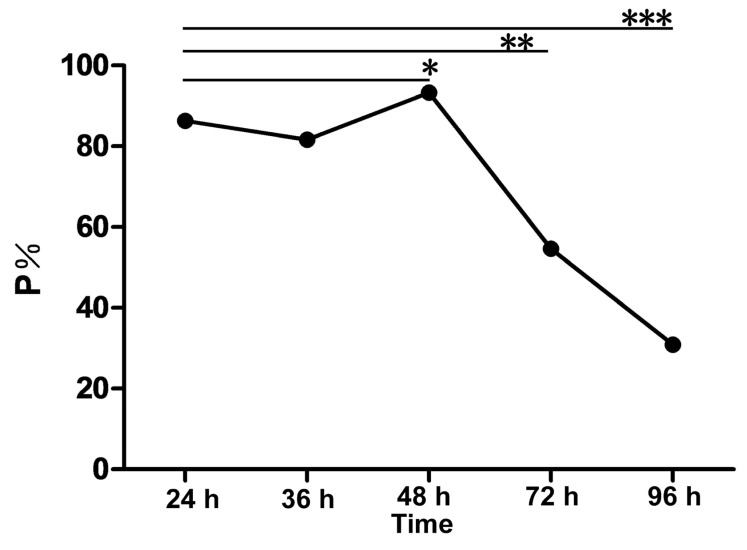
Proliferation of SV-HUC-1cells transfected with pcDNA3.1(+)/MgPa was detected by MTT (*n* = 3). The effect at the different time points of transfected with pcDNA3.1(+)/MgPa on cell proliferation is shown. Cell culture medium was planted in a 96-well plate overnight. After stimulating the cells, MTT solution was added to each well; later, formazan solution was added. Finally, absorbance was determined at 495 nm wavelength. Note: cell proliferation rate (P%), setting different groups (experimental group, EG; control group, CG) and culture medium as the zeroing group (ZG). P% = × (OD EG−ZG)/× (OD CG−ZG) × 100%. Note: * represents *p* < 0.05, ** represents *p* < 0.01 and *** represents *p* < 0.001.

MTT assays were used to further explore the effects of the interaction between rMgPa and RPL35 on SV-HUC-1 cells proliferation. As shown in Figure 7, the cell proliferation levels were increased at 24, 36, and 48 h after pcDNA3.1(+)/MgPa was transfected into cells, but this proliferation was inhibited at 72 h after the transfection. Hence, the interaction between rMgPa and RPL35 could promote cell proliferation at an early stage of *M. genitalium* infection.

## 3. Discussion

*Mycoplasma genitalium* was first isolated from the urethral secretions of a patient with nongonococcal urethritis and can cause urogenital infection. The bacterium has been linked to the death of patients suffering from HIV infection [17]. The adhesion of *M. genitalium* to host cells is the key to its colonization and subsequent invasion. MgPa is one of the most important adhesion proteins located mainly in the terminal organelle of *M. genitalium. Mycoplasma genitalium*’s process of infection or invasion into host cells is mediated by its adhesion proteins, which make it capable of causing damage to and the death of host cells. The essence of this process is the interaction between *M. genitalium* and the host cells. Ueno et al. demonstrated that *M. genitalium* can invade cervical carcinoma epithelial cells and non-cancerous endometrial cells (EM42) [18]. *Mycoplasma genitalium* penetrates Vero cells through its terminal organelle and colonizes its membrane-binding follicles [19]. Baseman et al. verified that *M. genitalium* survives up to 7 days in mammalian cells [20]. *Mycoplasma genitalium* can also replicate its DNA in host cells and increase its population [21].

However, few studies on the interaction between *M. genitalium* and host cells have been carried out. Based on the persistent infection caused by *M. genitalium* in vivo, there may be a mechanism that is beneficial to the long-term survival of *M. genitalium* in the host cells. Therefore, it is necessary to clarify how *M. genitalium* interacts with host cells and its survival mechanism after invading into the host cells. Researchers can insert a target DNA fragment into a phage genome to form a new fusion gene and display it on the surface of a phage using a phage-display technique [22]. Proteins or molecules of interest can be screened from phage-displayed libraries via several rounds of biopanning. At the same time, the non-specific phages can be removed by negative panning. Finally, the phages that bind to the specific target molecule can be obtained. Compared with other bacteriophages, the T7 phage particles exhibit high stability under various extreme conditions, including high temperature and low PH, thereby achieving efficient high-throughput affinity elution [23]. T7 phage-display system is mainly used in antigen epitope screening, vaccine development, protein interaction, and cancer diagnosis and treatment. For example, T7 phage display has been used to screen K-Ras(G12D)-selective inhibitor, which can inhibit A427 cancer cell proliferation [24]. Talwar et al. identified 10 highly significant *Mycobacterium tuberculosis* (TB) clones of smear-positive pulmonary tuberculosis patients by using the T7 phage-display technique. This can be used as an immunogenic antigen of therapeutic or prophylactic vaccines and as a target for tuberculosis treatment [25].

Our previous study confirmed that cyclophilin A (CypA), the key player for etiological agent’s infection, is the receptor protein of MgPa, which can partially inhibit *M. genitalium* from adhesion and even invasion into human urethral epithelial cells [16,26]. Our previous study also validated that MgPa can stimulate the secretion of CyPA in SV-HUC-1 cells and, thus, promote the expression of inflammatory cytokines through the CyPA–CD147–ERK–NF-κB signal pathway [27]. However, the specific MgPa protein may have many different interacting proteins. It is not hard to understand, plasminogen has more than one dozen plasminogen-interacting proteins [28]. It is possible to screen out different interacting proteins using different methods. Although the phage-display technique we used did not show that CyPA could interact with MgPa, the reason may be that we selected a relatively limited number of plaques (*n* = 50), which may be made up by expanding the sample size. However, the RPL35 we screened was present in the mass spectrometry sequencing of Deng et al. Differences in methods lead to some differences in research results, but this is not controversial. The interaction proteins screened by different methods will only enrich our research on the molecular function of MgPa. 

In this study, the T7 phage-displayed cDNA Library of SV-HUC-1 cells was successfully constructed and challenged using the purified rMgPa as a target molecule to screen the interacting protein of rMgPa from the whole genome of SV-HUC-1 cells. According to the results of DNA sequencing and homology analysis, seven representative bacteriophages that could specifically bind to rMgPa were screened and verified by phage ELISA and dot immunoassay. Results from far-Western blot and co-localization assays further confirmed that 60S ribosomal protein L35 (RPL35) from SV-HUC-1 cells could interact with rMgPa. Therefore, this study confirmed that RPL35 may be another interacting protein of MgPa besides CypA. This indicated that different MgPa interacting proteins may play different physiological roles during different processes of *M. genitalium* infection.

To investigate the effect of the interaction between rMgPa and RPL35 on cell function, TMT protein quantitative analysis was performed to screen differentially expressed proteins before and after pcDNA3.1(+)/MgPa was transfected into SV-HUC-1 cells. TMT is an in vitro chemical labeling reagent that can specifically label peptides produced by the enzymatic hydrolysis of proteins [29]. Because of its high efficiency and ability to label the ends of all peptides, TMT can provide a basis for the quantitative analysis of proteins. Therefore, it was used widely in the study of the pathogenesis of some diseases and biomarkers [30]. Crozier et al. identified hundreds of proteins related to the cell cycle regulation of *Trypanosoma brucei* using TMT analysis [31]. 

Based on the TMT technique, important differential proteins were screened after pcDNA3.1(+)/MgPa was transfected into cells in this study. The results showed that 401 proteins were upregulated, while 6 proteins were downregulated, with most of the upregulated proteins playing roles in the transcriptional initiation and translation of proteins, such as the SRP-dependent co-translation protein targeting of members (GO: 0006614), ribosome (GO:0005840), RNA binding (GO:0044822), and the main KEGG pathway including ribosome (hsa03010), which indicated that the interaction between MgPa and RPL35 could promote the transcription and translation of cellular proteins. In terms of cell composition, these upregulated proteins were mainly ribosome-related proteins, which suggested that the interaction between rMgPa and RPL35 could upregulate the expression of ribosome-related proteins. Representative factors associated with co-translation, translation initiation, ribosomal assembly, and mRNA transcription, including epidermal growth factor (EGF), eukaryotic translation initiation factor 2 (EIF2), signal recognition particle subunit 68 (SRP68), plasminogen activator inhibitor 1 RNA-binding protein (SERBP1), ribosomal protein L35A (RPL35A), and transforming growth factor beta-1-induced transcript protein (TGF-β) were selected for additional analysis. The mRNA expressions of these proteins were increased significantly after pcDNA3.1(+)/MgPa was transfected into cells, which suggested that the interaction between rMgPa and RPL35 could promote the transcription and translation of these proteins. These results were consistent with those of the TMT analysis.

RP, a major component of the ribosome, plays an important role in intracellular protein biosynthesis [32]. The ribosome-independent functions of RP include the regulation of cell proliferation, differentiation, and apoptosis [33]. RP is also closely related to tumorigenesis [34]. Interestingly, a growing number of studies have shown that RP has extracorporeal functions, based on which several criteria for determining RP-ribosomal extracellular functions have been proposed. Firstly, RP can interact with other non-ribosomal components inside the cell. Secondly, the interaction between RP and non-ribosomal components can lead to some physiological effects for cells. Thirdly, this physiological effect does not depend on the function of the ribosome itself but is independent of the ribosome [35,36]. In the process of ribosome synthesis, the dynamic balance between rRNA and RP needs to be achieved. Therefore, at a certain stage, some components of RP act as transcription inhibitors to regulate this balance. For example, RPL4 binds to a specific site of the promoter of the RPS10 gene and regulates the expression of RPS10 protein by inhibiting the initiation of RPS10 translation [37]. Singh et al. claimed that the activity of ribonuclease E could be regulated by the interaction of RPL4 and ribonuclease E, which leads to changes in mRNA composition during stress [38].

Researchers have found a strong link between *M. genitalium* infection and cancer. Miyake et al. and Erturhan et al. found that *M. genitalium* infection was associated with prostate cancer [39,40]. Idahl et al. found that *M. genitalium* infection was correlated with epithelial ovarian tumors [41]. *M. genitalium* is associated with tumorigenesis, but this correlation is not directly related to HIV infection, indicating that the cause of tumors induced by *M. genitalium* is also unclear. Our findings may provide some evidence, because we found abnormal cell proliferation after *M. genitalium* infection. Our results demonstrated that RPL35 was potentially the interacting protein of rMgPa, and the ensuing interaction could promote the proliferation of SV-HUC-1 cells. Therefore, we speculate that RPL35 acts as a transcription inhibitor at the early stage of *M. genitalium’s* invasion of host cells. This inhibition can be relieved when rMgPa binds to RPL35. The increase in protein translation levels can stimulate cell proliferation and provide nutrition for the growth and development of *M. genitalium* at the early stage of infection, which might be an important mechanism for *M. genitalium* to survive and colonize host cells during early infection. When *M. genitalium’s* colonization time in host cells is prolonged, the balance between rRNA and RP is broken, and the homeostasis of the intracellular environment is destroyed. Hence, the transcription and translation of proteins in epithelial cells are blocked, which leads to cell death, thus, exerting the bacterium’s pathogenicity on host epithelial cells. In future, we intend to study the effect of rMgPa and RPL35 interaction on cell function, and further assess the possible mechanism of the interaction between *M. genitalium* and RP to provide an experimental basis for understanding the survival strategies of *M. genitalium* and the biological function of rMgPa.

In conclusion, the results of this study demonstrated that RPL35 is the interacting protein of MgPa, and the interaction between MgPa and RPL35 can enhance the expression of transcription-initiation and translation-related proteins, and thus promote cell proliferation, which lays an experimental foundation for understanding the interaction between *M. genitalium* and host cells and the possible survival mechanisms of *M. genitalium.*

## 4. Materials and Methods

### 4.1. Chemicals and Reagents

The purification of recombinant *M. genitalium* protein of adhesion (rMgPa, 1075–1444 aa) using Ni-nitrilotriacetic acid (NTA) beads and the purification of the corresponding anti-rMgPa polyclonal antibody via affinity chromatography using CNBr-activated Sepharose 4B were performed as described in our previous study [42].

### 4.2. Preparation of the T7 Phage-Displayed cDNA Library of SV-HUC-1 Cells

The SV40 immortalized human urothelium cells (SV-HUC-1) (ATCC, CRL-9520) containing the SV40 genome were purchased from the Chinese Academy of Sciences Cell Bank (Shanghai, China). The preparation of the T7 phage-displayed cDNA library of SV-HUC-1 cells was performed as described by Luo et al. [43]. Approximately 150 μg of the total RNA of SV-HUC-1 cells was isolated using the nucleic acid isolation kit (Invitrogen, AM1975, Carlsbad, CA, USA). Highly purified mRNA was isolated by three rounds of purification using Magnetight Oligo (dT) particles by following instructions provided by the manufacturer (Promega, Madison, WI, USA). The T7 phage-displayed cDNA library of SV-HUC-1 cells was constructed using the T7 Select 10-3b cloning system (Novagen, Madison, WI, USA) as described by manufacturer. Briefly, the synthetic steps of reverse transcription (RT) from RNA to cDNA refer to the description of the reversed transcriptase box (Beyotime Biotech, Shanghai, China). The cDNA fragments were then connected with a T7 10-3b carrier arm at 16 °C overnight, after which a 5 μL ligation product was added to a 25 μL T7 select packaging extract protein for packaging in vitro. Finally, the packaging reaction was terminated by adding LB medium overnight at 37 °C. The phage clones were selected randomly for the amplification of the exogenous DNA sequence by PCR using 5′-GGAGCTGTCGTATTCCAGTC-3′ and 5′-AACCCCTCAAGACCCGTTTA-3′ as the forward and reverse primers, respectively, to assess the diversity of the library. The detection of titers for the SV-HUC-1 T7 phage-displayed cDNA library was shown in Supplementary Figure S1.

### 4.3. Biopanning and Analysis

The biopanning of T7 phage-displayed cDNA library of SV-HUC-1 cells was performed as described by Wang et al. [44]. Briefly, a microplate was coated using 100 μg/mL rMgPa. The microplate was kept overnight at 4 °C, and then the nonspecific sites were blocked the next day with 5% blocking reagent (skimmed milk) for 2 h at 4 °C. Subsequently, 200 µL of T7 phage-displayed cDNA library (3.2 × 10^8^ pfu) was added into the wells of the microplate and incubated for 60 min at 37 °C. The wells were then washed six times with Tris-HCl containing 0.1% Tween-20 (TBST) to remove the unbound phages. The bound phages were eluted with 200 µL of a T7 high salt elution buffer (1 M NaCl, 20 mM Tris-HCl, 2 mM KCl, 1 mM EDTA) and then were amplified in *Escherichia coli* BLT5403. The amplified phages were then subjected to three consecutive rounds of biopanning as described above, to enrich rMgPa-specific phages. 

Phage titers were measured using plaque assay as described in the Novagen T7 Select™ manual. Thirty-two plaques were randomly selected, and PCR was performed at 94 °C for 10 min, 50 °C for 1 min, 72 °C for 1 min, for 30 cycles and finally extended at 72 °C for 10 min using T7 primers as described above. 

### 4.4. Phage ELISA and Dot Immunobinding Assay

Representative phages were amplified with *E. coli* BLT5403 for further ELISA analysis. Ninety-six-well plates were coated with 100 µL of rMgPa. Next, 150 µL of amplified phages (approximately 3 × 10^8^ pfu) were added to each well, and incubated at 37 °C for 2 h, washed with TBS six times, subjected to 100 µL (per well) of diluted T7 tail fiber monoclonal antibody (Cat. bs-2107R, Bosen Co., Ltd, Guangdong, China), and incubated for 1 h at room temperature. The unbound T7 tail fiber monoclonal antibody was removed with TBST after the incubation process. Then, 100 µL of diluted HRP-conjugated goat anti-rabbit IgG antibody (ab6721, Abcam, Cambridge, UK) was introduced into each well, and the mixture was left to incubate for 1 h at room temperature. The plates were washed with TBST at the end of the incubation period. In the final step, the contents of the microplate were colored for observation at 37 °C for 15 min using a tetramethylbenzidine (TMB) solution as a substrate. After adding H_2_SO_4_ (2M), the optical density (OD) value for each well was obtained at 450 nm using a multifunctional microplate reader, Infinite^®^ F50 (Mannedorf, Switzerland). 

A dot immunobinding assay for phages was performed as described by Ali [45]. Briefly, each representative phage (approximately 3 × 10^8^ pfu) was tested on each grid of a poly vinylidene fluoride (PVDF) membrane, followed by drying of the PVDF membrane at room temperature and blocking with skim milk (0.5%) at 4 °C overnight. The PVDF membrane was incubated with 100 µg/mL of the rMgPa protein in a warm box for 2 h and then washed six times. Following the 2 h incubation, a primary antibody (rabbit anti-rMgPa antibody) was added, and the reaction was left for another 2 h incubation at 37 °C. The membrane was then washed five times, and a third incubation was performed, this time with a secondary antibody (HRP-labeled goat anti-Rabbit IgG antibody) for 1 h at 37 °C. Finally, the membrane was washed five times, colored with TMB (Beyotime, Shanghai, China) for observation, and then developed and fixed.

### 4.5. Interaction Assays between rMgPa and RPL35 Using Far-Western Blotting

The extraction of total protein from SV-HUC-1 cells was performed as described by Deng et al. [16]. Briefly, when the SV-HUC-1 cells were about 90% confluent, cells were digested using trypsin and centrifuged at 1000 rpm for 6 min at room temperature. Then, 100 µL of RIPA (50 mM Tris-HCl, pH7.4, 150 mM NaCl, 1% NP-40) and 100 μg/mL of PMSF was added, and the reaction mixture was allowed to incubate in the icebox for 30 min. The resulting content was subjected to centrifugation at 12,000 rpm for 15 min at 4 °C. The supernatant, namely the protein of interest, was collected and stored at −20 °C. 

The interaction between rMgPa and RPL35 was identified from total cell proteins using far-Western blotting as described by Fecková et al. [46]. Briefly, 10 µg of rMgPa was separated by 12% of SDS-PAGE and transferred to a PVDF membrane using a semi-dry transfer unit. After blocking with 5% skim milk overnight at 4 °C, the PVDF membrane was incubated with total cell proteins (1:500) for 6 h at 4 °C. A second incubation of the PVDF membrane followed with a rabbit anti-RPL35 antibody (1:200) (Cat. GR303483-1, Abcam, Cambridge, UK) for 2 h at 37 °C. The membrane was then washed six times with TBST, incubated with an HRP-conjugated goat anti-rabbit IgG antibody (1:4000) for 2 h at 37 °C, and washed six times with TBST. A chemiluminescent imaging system (Syngene, Frederick, MD, USA) was used to detect immunoreactive bands on the membrane. 

### 4.6. Co-localization Analysis of rMgPa and RPL35 in the SV-HUC-1 Cells

Co-localization analysis of rMgPa and RPL35 was performed as described by Deng et al. The MgPa target gene was amplified using 5′-ggaattcatgcctaaatcactgtgggatc-3′ and 5′-ccgctcgagcactactataggaacagtt-3′ as up and down primers, respectively. The conditions for amplification were predenaturation at 94 °C for 5 min, denaturation at 94 °C for 30 s, annealing at 52 °C for 45 s, extension at 72 °C for 1 min 20 s for 35 cycles, and extension for 7 min at 72 °C. PCR products were then digested with *EcoR* I and *Xho* I (Thermo Science, Waltham, MA, USA) before being cloned into the corresponding restriction sites of the pcDNA3.1(+) vector to construct the recombinant vector pcDNA3.1(+)/MgPa (Supplementary Materials Figure S3). The expression of the rMgPa proteins as detected by Western blotting is shown in Supplementary Materials Figure S4. The pcDNA3.1(+)/MgPa vector was transfected into SV-HUC-1 cells. The transfected cells were prepared and incubated with a primary antibody mixture containing a rabbit anti-rMgPa antibody (1:1000) and a mouse anti-RPL35 antibody (Cat. H00011224-A01, Invitrogen, AM1975, Carlsbad, CA, USA), washed six times with TBST, and then incubated with a secondary antibody mixture containing a FITC-conjugated AffiniPure goat anti-mouse IgG (H + G) (1:200) (Cat. CW0152S, Beyotime, Shanghai, China) and a TRITC-conjugated AffiniPure goat anti-rabbit IgG (H + L) (1:200) (Cat. CW0114S, Beyotime, Shanghai, China) for 2 h at 37 °C. After washing the samples six times, 200 µL of DAPI was added for the staining of nuclei. Staining with DAPI caused the delineation of nuclei of SV-HUC-1 cells, helping identify their intracellular planes. The exposure time was concordant for all images in each set of the experiment, and the images were viewed under an inverted TE2000-S microscope (Nikon, Tokyo, Japan).

### 4.7. Analysis of the Transcriptome of SV-HUC-1 Cells by TMT Analysis

The total cellular protein was extracted from the pcDNA3.1(+)/MgPa-transfected and the empty-plasmid-transfected SV-HUC-1 cells, respectively, using 300 µL of a RIPA (0.1% PMSF) lysate. TMT analysis was carried out by the Abace Biotechnology Company (Beijing, China). Differential proteins were sorted out and classified, with 0.05 considered the significant threshold. Selecting *Homo sapiens* as the background and species, the gene ontology (GO) information of altered protein expression was analyzed for biological processing (BP) and cell composition (CC). 

### 4.8. RT-qPCR Analyses of the mRNA Expression of Differential Protein 

Total cellular RNA was isolated from SV-HUC-1 cells using 500 µL of Trizol reagent (Beyotime Biotech, Shanghai, China) according to the manufacturer’s recommendations. Based on the results of the TMT analyses, real-time quantitative reverse transcription PCR (RT-qPCR) was used to detect the RNA level of proteins associated with cell transcription and translation functions. The primer sequences of these proteins were shown in Table 3. The synthetic steps for reverse transcription from RNA to cDNA were performed using the FastQuant cDNA first-strand synthesis kit (Beyotime, Shanghai, China). Total RNA (1 µg) was incubated for 60 min at 42 °C and 10 min at 80 °C. The cDNA samples from RT reactions were amplified using the 2× Super Real PreMixplus MasterMix (Tiangen Biotech Co., Ltd., Beijing, China). The thermal cycling conditions were as follows: 95 °C for 5 min, followed by 40 cycles at 95 °C for 15 s, 58 °C for 20 s, and 72 °C for 15 s using Roche LightCycler^®^ 96.

### 4.9. The Detection of Cell Proliferation Using the MTT Assay 

MTT assays were used to evaluate the survival of cells in different groups. The EG included the pcDNA3.1 (+)/MgPa-transfected cells, the CG the empty plasmid pcDNA3.1 (+) cells, and the ZG the medium treatment group, and the ZG contained PBS. Ten microliters of MTT (Beyotime, China) was added into a 96-well plate. After an additional incubation at 37 °C and 5% CO_2_ for 4 h, 100 μL of dimethyl sulfoxide was added into each well. After an additional incubation at 37 °C, 5% CO_2_ for 4 h, 100 μL dimethyl sulfoxide was added into each well. Finally, the absorbance was measured at 495 nm using a multifunctional Microplate Infinite^®^ F50 reader.

### 4.10. Statistical Analysis

All statistical analysis were conducted using the GraphPad Instant statistical package (GraphPad Software Inc., San Diego, CA, USA), and data were analyzed using the SPSS18.0 statistical software. The data are expressed as mean ± standard deviation. The criterion for statistical significance was set at *p* < 0.05 and *p* < 0.01.

## Figures and Tables

**Table 1:** The input, output, and ratio for biopanning of library.

Rounds	First	Second	Third	Fourth
Input (pfu)	3 × 10^8^	3 × 10^8^	3 × 10^8^	3 × 10^8^
Output (pfu)	2 × 10^4^	4.2 × 10^4^	1.9 × 10^5^	5 × 10^5^
Ratio	0.67 × 10^−4^	1.4 × 10^−4^	6.3 × 10^−4^	1.7 × 10^−3^

**Table 2:** BLAST analysis for exogenous DNA sequences of positive phages.

Number	Coded Protein	mRNA ID	Matched Sequence Range	Similarity	Repeated Times
P1	RPL35	NM_007209.4	102–196 bp	100%	20
P2	Mitochondrial complete genome	NC_012920.1	1312–1600 bp	99%	3
P3	RTN4	NM_020532.5	391–490 bp	100%	3
P4	COX6AI	NM_004373.4	15–344 bp	100%	2
P5	RPL21	NM_000982.4	50–494 bp	99%	2
P6	RPS23	NM_001025.5	32–453bp	99%	1
P7	RPS26	NM_001029.5	250–597bp	99%	1
Total					32

Note: Homological analysis results for the inserted exogenous DNA sequences of 32 phages; the 7 representative phages were numbered as P1 to P7 (*n* = 32).

**Table 3:** Primer sequences of the target genes.

Gene	Primer Sequence (5′–3′)
*GAPDH*	F: 5′-GCACCGTCAAGGCTGAGAAC-3′ R:5′-TGGGAAGACGCCAGTGGA-3′
*EIF2*	F:5′-CGAGAAGCACAGCAAGAACATCAC-3′ R:5′-TCCTACAGACGCCTTCTCTTCGG-3′
*SRP68*	F:5′-CTCTCGCACCTGGTCTCCTACG-3′ R:5′-GCTCCAACACGCTGCCACTG-3′
*SERBP1*	F:5′-AAGAGGCTCATGCTGAAGATTCGG-3′ R:5′-AGGAGCAGAAGCACTTGACTTGTC-3′
*RPL35A*	F:5′-TGGAAGGCTGTGGTCCAAGGC-3′ R:5′-CGCCAGGAGTGACTGTGTTGTTC-3′
*EGF*	F:5′-ATGGCCAATCTGGATGGTTC-3′ R:5′-CATGCTGCCTTGGAGACGTA-3′
*TGF-β*	F:5′-GTGAAACACCGAGGACACCT-3′ R:5′-GGTGCGTTGATAAATGTGG-3′

## Data Availability

Data are available on reasonable request to the corresponding author.

## Supplementary Materials

The following are available online at https://www.mdpi.com/article/10.3390/pathogens10111449/s1, Figure S1: The detection of titer for SV-HUC-1 T7 phage display cDNA library, Figure S2: Agarose gel electrophoresis analysis of PCR products of the displayed phages, Figure S3: Construction of pcDNA3.1(+)/rMgPa. Mycoplasmal TGA codons were substituted with TGG (3223~4092aa), Figure S4: Expression of the rMgPa proteins as detected by western blotting, Figure S5: Blast mapping of BC071915.1, BC094828.1 and BC000348.2 of RPL35 subunits with exogenous sequences.
