# Supplementary material for: Mycoplasma genitalium Protein of Adhesion Promotes the Early Proliferation of Human Urothelial Cells by Interacting with RPL35
Source: Pathogens. 2021 Nov 8;10(11):1449. doi: 10.3390/pathogens10111449 (PMC8621731; doi:10.3390/pathogens10111449)
Supplement: Supplementary file 1 [file pathogens-10-01449-s001.zip › pathogens-1406832-supplementary.pdf]

Supplementary

Mycoplasma genitalium protein of adhesion promotes the early proliferation of human urothelial cells by interacting with RPL35

Pei Dai, Xiangying Deng, Peng Liu, Lingling Li, Dan Luo, Yating Liao, Yanhua Zeng*

Institute of Pathogenic Biology, Hengyang Medical College, University of South China; Hunan Provincial Key Laboratory for Special Pathogens Prevention and Control; Hunan Province Cooperative Innovation Center for Molecular Target New Drug Study, Hengyang 421001, P.R. China;

* **Corresponding author.**

Yanhua Zeng: Institute of Pathogenic Biology, Hengyang Medical College, University of South China; Hunan Provincial Key Laboratory for Special Pathogens Prevention and Control; Hunan Province Cooperative Innovation Center for Molecular Target New Drug Study, Hengyang 421001, P.R.China.

E–mail: zengyihua21cn@usc.edu.cn

Running title: The interaction between the *Mycoplasma genitalium* protein of adhesion and RPL35.

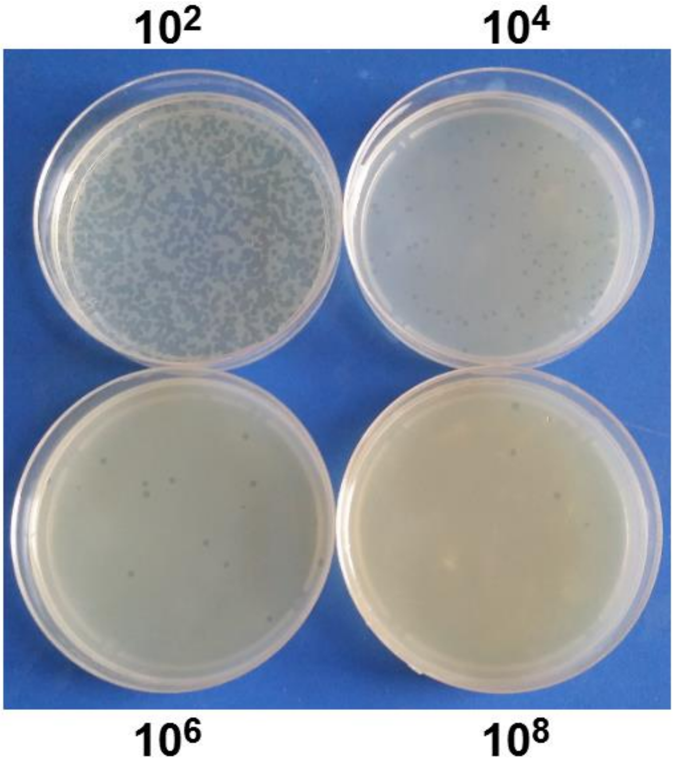

Supplementary Figure S1. The detection of titer for SV-HUC-1 T7 phage display cDNA library. The SV-HUC-1 T7 phage display cDNA library constructed by our research group was amplified in the host strain BLT5403. The phage library was diluted into different multiples and cultured overnight in 37°C. incubator in LB-SRB medium containing Carbenicillin. The calculated titer is 3×10^8pfu. The result is shown in figure 1. Lanes: The 10^2 , 10^4 , 10^6 and 10^8 represents different phage dilution times.

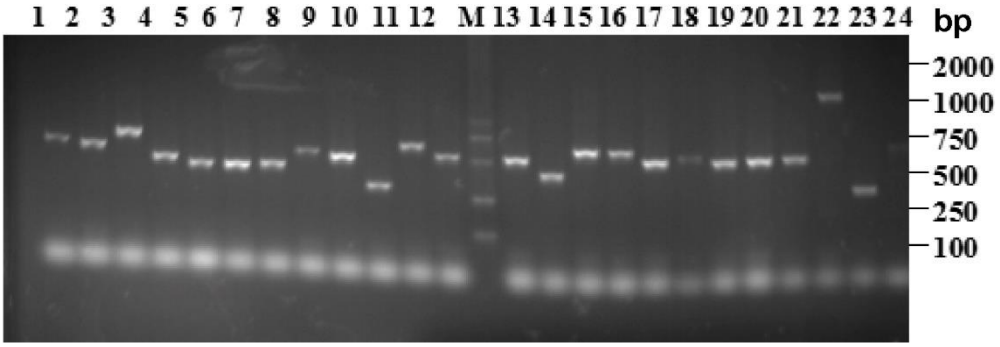

Supplementary Figure S2. Agarose gel electrophoresis analysis of PCR products of the displayed phages. After four rounds of panning, the final enriched specific clones were plated and single pure plaque was isolated. The cDNA inserts in these plaques were amplified by PCR using primers and 50 clones were randomly picked and out of which 32 clones got clear amplified products and Agarose gel electrophoresis analysis of PCR products for partial phages as figure 2 showed. Note: M, DNA marker; Lane1~24, Number of different phage plaques.

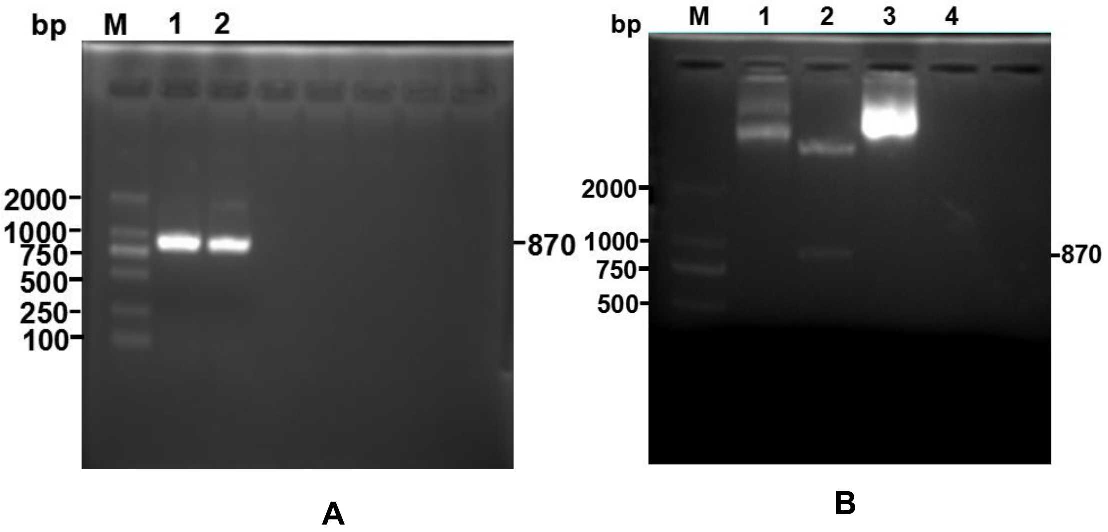

Supplementary Figure S3. Construction of pcDNA3.1(+)/rMgPa. Mycoplasmal TGA codons were substituted with TGG (3223~4092aa). *Xho*I and *EcoR*I restriction enzymes were purchased from Thermofisher Scientific). According to the primer design guidelines (Sangon Biotech Co, Ltd, Shanghai, China). **(A)** Agarose gel electro phoresis analysis of PCR products of MgPa. Note: M, DNA marker; Lane1-2, PCR product of MgPa. **(B)** The recombinant plasmid was verified by double-enzyme cleavage. Note: M, DNA marker; Lane1, pcDNA3.1(+)/rMgPa Products; 2, pcDNA3.1 (+) /rMgPa was digested with *EcoR*I and *Xho*I; 3, pcDNA3.1 (+) null plasmid.

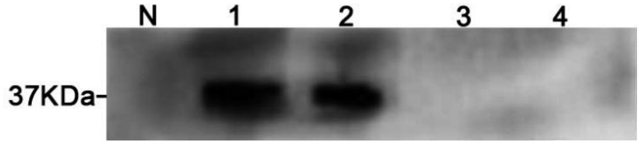

Supplementary Figure S4. Expression of the rMgPa proteins as detected by western blotting. The western blotting results revealed that rMgPa was detected in SV-HUC-1 cells transfected with pcDNA3.1(+)/rMgPa. By contrast, no rMgPa expression was observed in control groups. Note: N, Normal; Lane1and Lane2 transfection of SV-HUC-1cells with pcDNA3.1(+)/rMgPa. lane3, pcDNA3.1(+); Lane4, lip2000 (invitrogen). There was a distinct band observed at approximately 37kDa such as 1 and 2, whereas no band was observed for the control group as N, 3 and 4.

Homo sapiens ribosomal protein L35, mRNA (cDNA clone MGC:88597 IMAGE:4896387), complete cds

Sequence ID: BC071915.1 Length: 457 Number of Matches: 1

Range 1: 1 to 445 GenBank Graphics ▼ Next Match ▲ Previous Match

Score	Expect	Identities	Gaps	Strand
817 bits(442)	0.0	444/445(99%)	0/445(0%)	Plus/Plus

```
Query  36   GCGGCTTGTGCAGCAATGGCCAAGATCAAGGCTCGAGATCTTCGCGGGAAGAAGAAGGAG  95
            ||||||||||||||||||||||||||||||||||||||||||||||||||||||||||||
Sbjct  1    GCGGCTTGTGCAGCAATGGCCAAGATCAAGGCTCGAGATCTTCGCGGGAAGAAGAAGGAG  60

Query  96   GAGCTGCTGAAACAGCTGGACGACCTGAAGGTGGAGCTGTCCCAGCTGCGCGTCGCCAAA  155
            ||||||||||||||||||||||||||||||||||||||||||||||||||||||||||||
Sbjct  61   GAGCTGCTGAAACAGCTGGACGACCTGAAGGTGGAGCTGTCCCAGCTGCGCGTCGCCAAA  120

Query  156  GTGACAGGCGGTGCGGCCTCCAAGCTCTCTAAGATCCGAGTCGTCCGGAAATCCATTGCC  215
            ||||||||||||||||||||||||||||||||||||||||||||||||||||||||||||
Sbjct  121  GTGACAGGCGGTGCGGCCTCCAAGCTCTCTAAGATCCGAGTCGTCCGGAAATCCATTGCC  180

Query  216  CGTGTTCTCACAGTTATTAACCAGACTCAGAAAGAAAACCTCAGGAAATTCTACAAGGGC  275
            ||||||||||||||||||||||||||||||||||||||||||||||||||||||||||||
Sbjct  181  CGTGTTCTCACAGTTATTAACCAGACTCAGAAAGAAAACCTCAGGAAATTCTACAAGGGC  240

Query  276  AAGAAGTACAAGCCCCTGGACCTGCGGCCTAAGAAGACACGTGCCATGCGCCGCCGGCTC  335
            ||||||||||||||||||||||||||||||||||||||||||||||||||||||||||||
Sbjct  241  AAGAAGTACAAGCCCCTGGACCTGCGGCCTAAGAAGACACGTGCCATGCGCCGCCGGCTC  300

Query  336  AACAAGCACGAGGAGAACCTGAAGACCAAGAAGCAGCAGCGGAAGGAGCGGCTGTACCCG  395
            ||||||||||||||||||||||||||||||||||||||||||||||||||||||||||||
Sbjct  301  AACAAGCACGAGGAGAACCTGAAGACCAAGAAGCAGCAGCGGAAGGAGCGGCTGTACCCG  360

Query  396  CTGCGGAAGTACGCGGTCAAGGCCTGAGGGGCGCATTGTCAATAAAGCACAGCTGGCTGA  455
            ||||||||||||||||||||||||||||||||||||||||||||||||||||||||||||
Sbjct  361  CTGCGGAAGTACGCGGTCAAGGCCTGAGGGGCGCATTGTCAATAAAGCACAGCTGGCTGA  420

Query  456  GGaaaaaaaaaaaaaaaaaaaaaaa  480
            | |||||||||||||||||||||||
Sbjct  421  GAAAAAAAAAAAAAAAAAAAAAAAA  445
```

Sequence ID: **Query_44843** Length: **996** Number of Matches: **1**

Range 1: 49 to 408 Graphics **BC094828.1** ▼ Next Match ▲ Previous Match

Score	Expect	Identities	Gaps	Strand
593 bits(321)	7e-174	347/360(96%)	0/360(0%)	Plus/Plus

```
Query  1    GCAATGGCCAAGATCAAGGCTCGAGATCTTCGCGGGAAGAAGAAGGAGGAGCTGCTGAAA  60
            ||||||||||||||||||||||||||||||| ||||||||||||||||||||||||||||
Sbjct  49   GCAATGGCCAAGATCAAGGCTCGAGATCTTCCCGGGAAGAAGAAGGAGGAGCTGCTGAAA  108

Query  61   CAGCTGGACGACCTGAAGGTGGAGCTGTCCCAGCTGCGCGTCGCCAAAGTGACAGGCGGT  120
            ||||||||||||||||||||||||||||||||||||||||||||||||||||||||||||
Sbjct  109  CAGCTGGACGACCTGAAGGTGGAGCTGTCCCAGCTGCGCGTCGCCAAAGTGACAGGCGGT  168

Query  121  GCGGCCTCCAAGCTCTCTAAGATCCGAGTCGTCCGGAAATCCATTGCCCGTGTTCTCACA  180
            ||||||||||||||||||||||||||||||||||||||||||||||||||||||||||||
Sbjct  169  GCGGCCTCCAAGCTCTCTAAGATCCGAGTCGTCCGGAAATCCATTGCCCGTGTTCTCACA  228

Query  181  GTTATTAACCAGACTCAGAAAGAAAACCTCAGGAAATTCTACAAGGGCAAGAAGTACAAG  240
            |||||||||||||||| |||||||||||||||||||||||||||||||||||||||||||
Sbjct  229  GTTATTAACCAGACTCTTAAAGAAAACCTCAGGAAATTCTACAAGGGCAAGAAGTACAAG  288

Query  241  CCCCTGGACCTGCGGCCTAAGAAGACACGTGCCATGCGCCGCCGGCTCAACAAGCACGAG  300
            |||||||||||||||||||| || ||||||||||||| ||||| ||||||||||||||||
Sbjct  289  CCCCTGGACCTGCGGCCTAAAAACACACGTGCCATGCTCCGCCTGCTCAACAAGCACGAG  348

Query  301  GAGAACCTGAAGACCAAGAAGCAGCAGCGGAAGGAGCGGCTGTACCCGCTGCGGAAGTAC  360
            || |||||||||||||||||||||| ||||||||| ||| ||||||||||||| || |||
Sbjct  349  GACAACCTGAAGACCAAGAAGCAGCCGCGGAAGGATCGGATGTACCCGCTGCGAAACTAC  408
```

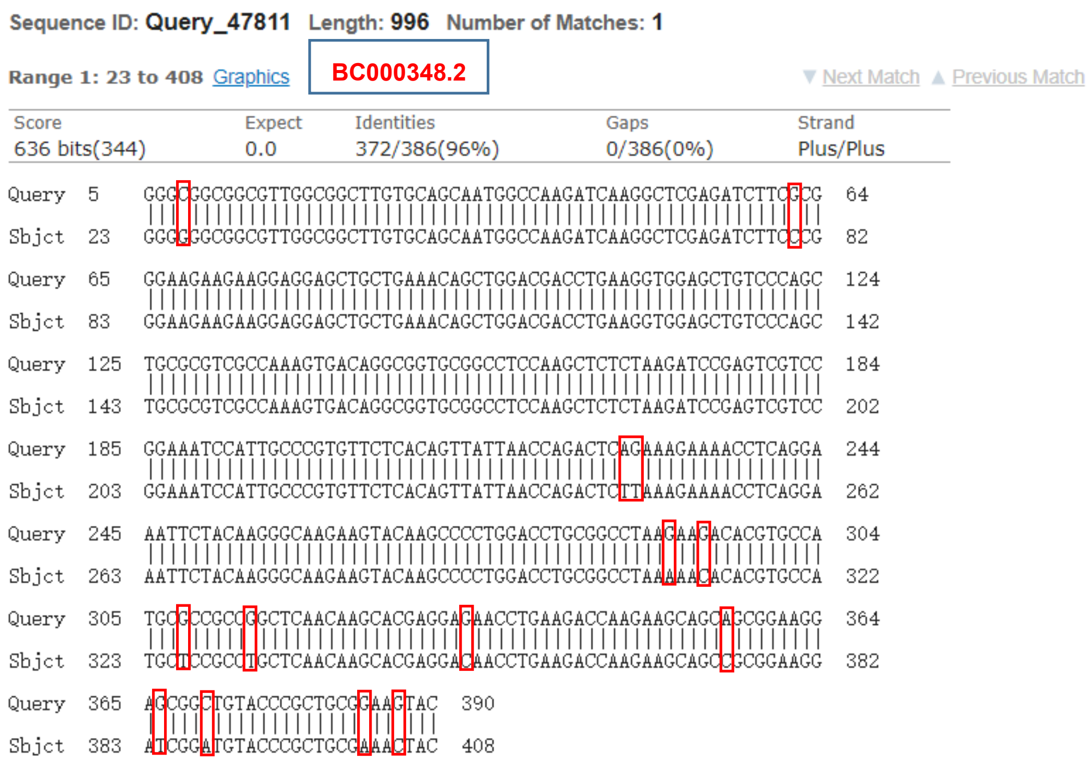

Supplementary Figure S5. Blast mapping of BC071915.1, BC094828.1 and BC000348.2 of RPL35 subunits with exogenous sequences.
